# Characterization of vector communities and biting behavior in South Sulawesi with host decoy traps and human landing catches

**DOI:** 10.1186/s13071-020-04205-z

**Published:** 2020-06-29

**Authors:** Jenna R. Davidson, Robert N. Baskin, Hajar Hasan, Timothy A. Burton, Muhammad Wardiman, Nur Rahma, Fadly R. Saputra, Muhammad Sultanul Aulya, Isra Wahid, Din Syafruddin, Frances M. Hawkes, Neil F. Lobo

**Affiliations:** 1 Eck Institute for Global Health, University of Notre Dame, Notre Dame, Indiana, 46556 USA; 2 Department of Parasitology, Faculty of Medicine, Hasanuddin University, Makassar, 90245 Indonesia; 3 Eijkman Institute of Molecular Biology, Jakarta, Indonesia; 4 Natural Resources Institute, University of Greenwich, Central Avenue, Chatham Maritime, Kent, ME4 4TB UK

**Keywords:** *Culex*, Host decoy trap, Surveillance, Sampling device, Behaviour, Indonesia, Arbovirus, Malaria

## Abstract

**Background:**

Indonesia has high mosquito diversity, with circulating malaria and arboviruses. Human landing catches (HLC) are ethically questionable where arboviral transmission occurs. The host decoy trap (HDT) is an exposure-free alternative outdoor sampling device. To determine HDT efficacy for local culicids, and to characterize local mosquito fauna, the trapping efficacy of the HDT was compared to that of HLCs in one peri-urban (Lakkang) and one rural (Pucak) village in Sulawesi, Indonesia.

**Results:**

In Lakkang the outdoor HLCs collected significantly more *Anopheles* per night (*n* = 22 ± 9) than the HDT (*n* = 3 ± 1), while the HDT collected a significantly greater nightly average of *Culex* mosquitoes (*n* = 110 ± 42), than the outdoor HLC (*n* = 15.1 ± 6.0). In Pucak, there was no significant difference in *Anopheles* collected between trap types; however, the HDT collected significantly more *Culex* mosquitoes than the outdoor HLC nightly average (*n* = 53 ± 11 *vs* 14 ± 3). Significantly higher proportions of blood-fed mosquitoes were found in outdoor HLC (*n* = 15 ± 2%) compared to HDT (*n* = 2 ± 0%). More blood-fed culicines were collected with outdoor HLC compared to the HDT, while *Anopheles* blood-fed proportions did not differ. For the HDT, 52.6%, 36.8% and 10.5% of identified blood meals were on cow, human, and dog, respectively. Identified blood meals for outdoor HLCs were 91.9% human, 6.3% cow, and 0.9% each dog and cat. Mosquitoes from Pucak were tested for arboviruses, with one *Culex* pool and one *Armigeres* pool positive for flavivirus, and one *Anopheles* pool positive for alphavirus.

**Conclusions:**

The HDT collected the highest abundance of culicine specimens. Outdoor HLCs collected the highest abundance of *Anopheles* specimens. Although the HDT can attract a range of different Asian mosquito genera and species, it remains to be optimized for *Anopheles* in Asia. The high proportion of human blood meals in mosquitoes collected by outdoor HLCs raises concerns on the potential exposure risk to collectors using this methodology and highlights the importance of continuing to optimize a host-mimic trap such as the HDT.
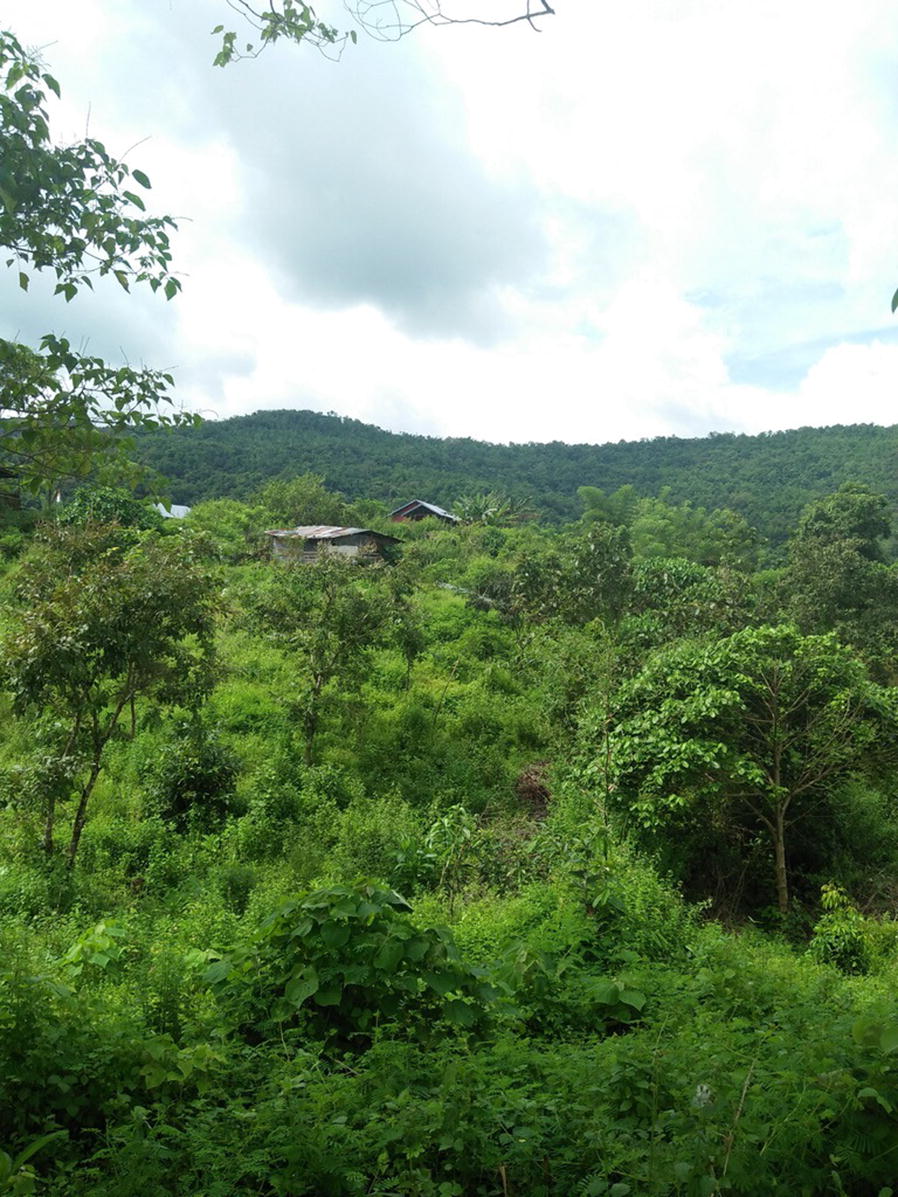

## Background

The Indonesian archipelago, located along the equator, is made up of over 17,000 islands [1] and is recognized as one of 17 mega-biodiverse countries on the planet [2, 3]. This biodiversity extends to insect vector communities; the archipelago has high *Anopheles* diversity, with 21 previously confirmed malaria vector species and species complexes [4–8]. Also widespread in Indonesia are *Aedes aegypti* and *Aedes albopictus*, the primary vectors of several important arboviruses, including dengue and chikungunya virus [9–12]. Numerous other *Aedes* species are present in Indonesia and many are capable of transmitting the causative agents of rift valley fever and filariasis [13–20]. Furthermore, species of the genus *Culex* are ubiquitous throughout much of Indonesia, with many identified as the vectors of filarial parasites, as well as a number of lesser known arboviruses [19, 21–27].

The Republic of Indonesia has the fourth largest population in the world and half of that population live in malaria endemic regions [28, 29]. Disease control relies heavily on vector control measures such as insecticide-treated nets (ITNs) and indoor residual spraying (IRS) [4]. Given the reliance on these vector control measures, the WHO recommends that malaria control programmes also implement entomological monitoring to assess their impact on vector populations, as well as possible changes in intervention efficacy resulting from either changes in mosquito behavior or the emergence of insecticide resistance [30]. The traditional method to monitor vector populations has been the human landing catch (HLC), which involves persons sitting with their lower legs exposed and collecting mosquitoes that come to feed on them during the night [31]. This is the most direct measure of mosquito biting and can be implemented indoors, outdoors, or at any site where transmission may occur, and provides several important entomological endpoints relevant to understanding local epidemiological outcomes. These entomological endpoints are critical data that allow disease control programmes to determine site-specific transmission dynamics and inform the design of evidence-based strategic intervention, as well as identify gaps in protection.

Although HLCs are the sampling method most indicative of vectors biting hosts, they have come under scrutiny due to ethical concerns around exposing collectors to infectious bites [32]. The WHO recommends universal ITN coverage for all persons living in malaria endemic areas [33, 34]; employing persons to stay up all night for the purpose of collecting host-seeking mosquitoes exposes them to malaria and/or other vector-borne diseases that they may have avoided if protected by an ITN. Though ethical concerns about malaria incidence in HLC collectors may be mitigated by two compelling studies that demonstrate no difference in infection rates in the community *versus* those conducting HLCs, as well as the positive impacts of prophylaxis [35, 36], there exists the risk of non-malarial arboviral disease transmission for which there is limited prophylaxis or treatment. Given that Indonesia is a biodiversity hotspot with multitudes of malaria and arboviral vectors, an ethically acceptable sampling method that targets a wide variety of bionomics, is cost effective and easily implemented is imperative to expand disease control and knowledge of vector populations. In the context of Indonesia’s high diversity of mosquito species, outdoor sampling is particularly important, as many vectors readily feed outdoors. While alternative indoor sampling methods have been developed for indoor environments, there are limited tools proven to be effective and versatile for attracting and collecting outdoor-biting vectors [37, 38].

The host decoy trap (HDT) represents an exposure-free and passive sampling device suitable for outdoor use that attempts to artificially recreate the stimuli that mosquitoes use when host-seeking and feeding [39]. By mimicking visual, olfactory, and thermal stimuli of natural mosquito hosts, an HDT can be used for sampling and killing mosquitoes. Furthermore, these stimuli are all easily manipulated, making the HDT highly versatile for targeting species with known host preferences. When tested outdoors in Burkina Faso, HDTs collected *Anopheles coluzzii*, a typically indoor-feeding vector, at a ratio of nearly ten to one compared to outdoor human landing catches [39]. It was also found that regardless of season or mosquito genera, HDT catches always outnumbered HLC [39]. More recently, HDTs were evaluated in Kisian and Orego, two villages in western Kenya, to assess its performance in attracting exophagic and zoophagic vectors of malaria using human and cow odors [40]. The study found that a cattle-baited HDT consistently caught more anophelines outdoors than an HLC [40]. However, The Kisian village results contrasted with the original study, as human-baited HDTs caught a lower number of anophelines than HLCs. Local vector populations in western Kenya differ to those in Burkina Faso, and thus the HDT’s efficacy may be linked to species-specific differences in vector behaviour and bionomics. Although HDTs have been evaluated using field studies in Africa, focusing on sampling of the *Anopheles gambiae* (*sensu lato*) species complex, this tool has not been evaluated as a sampling method in Asia, where it could provide malaria and arboviral endemic regions with a human exposure-free sampling device for entomological investigations [39, 40].

To the best of our knowledge, this study is the first evaluation of the human decoy trap outside of Africa, representing the first field test of the behavioural principles employed in the design of the HDT on Asian (Indonesian) mosquito fauna. Due to its unique geological history and stable year-round climate, Sulawesi, the largest island of Wallacea, has particularly elevated levels of species richness and endemism [41–43] and was therefore chosen as the location for the study. Furthermore, no studies surveying the local mosquito compositions or bionomics in Sulawesian areas of Lakkang or Pucak villages have been published. Our aims were to (i) evaluate the efficacy of the HDT trap relative to outdoor HLCs with respect to species catch frequency, abdominal status, and blood meal hosts and (ii) establish basic information regarding species composition, host preferences, and flight activity in Lakkang and Pucak villages in Sulawesi, Indonesia.

## Methods

### Study setting

#### Lakkang

**Fig. 1:**
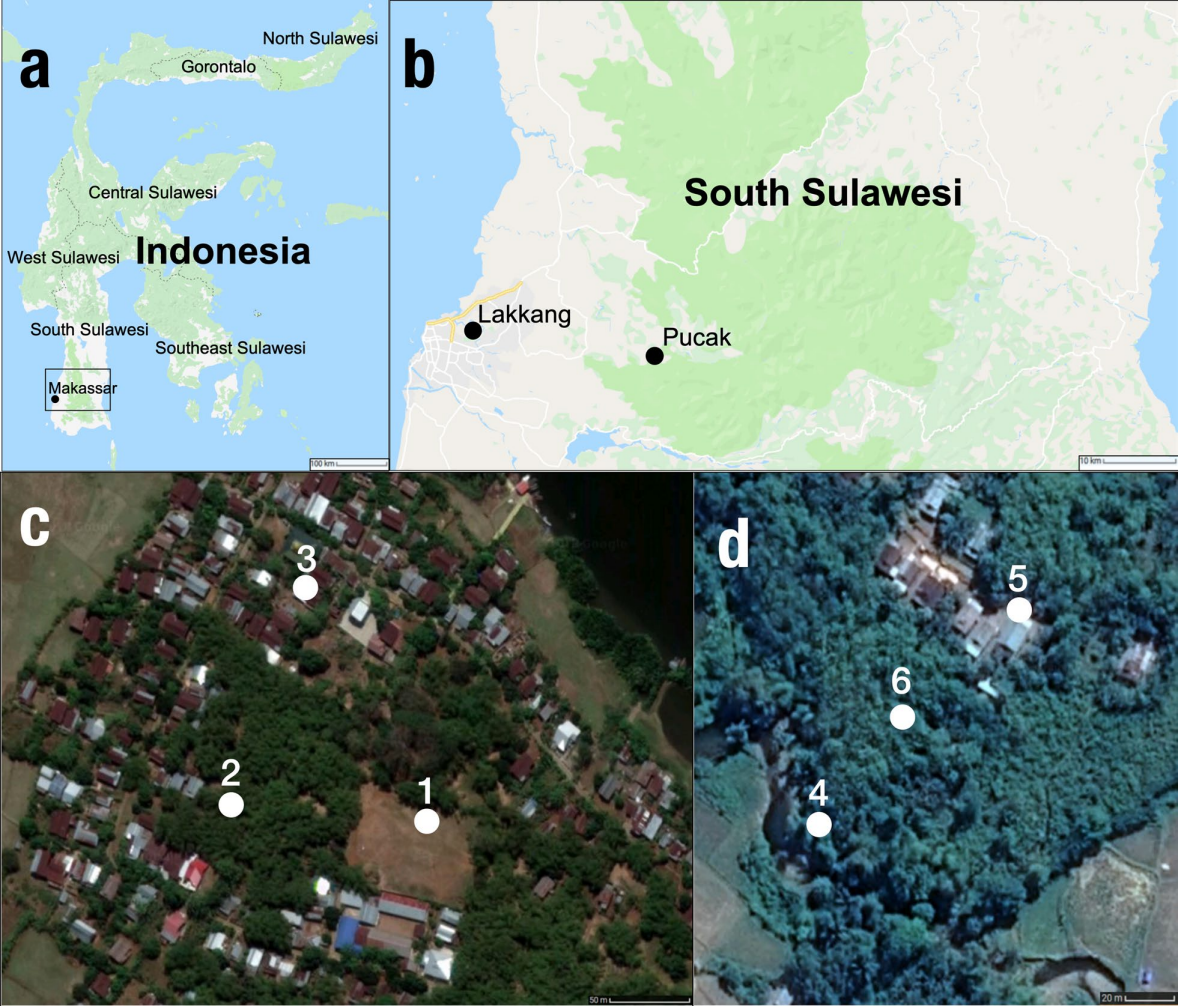
Study locations and collection sites in South Sulawesi, Indonesia. **a** Sulawesi, Indonesia. **b** Lakkang and Pucak village locations in South, Sulawesi, Indonesia. **c** Collection sites one, two, and three in Lakkang. **d** Collection sites four, five, and six in Pucak

Located in Tallo, Makassar Regency of South Sulawesi, Lakkang Village is 3.9 km from Makassar city center. However, this peri-urban village is surrounded by rivers, making the isolated location distinct from its large neighboring city (Fig. 1a–c). The village covers approximately 1 km^2^ and has a population of approximately 1200 inhabitants, primarily engaged in rice farming and fishing. Typical dwellings here are based on an open concept two-story wood and plaster construction with metal roof and open-air access to outdoors much of the day and evening. Lakkang is considered to be malaria receptive. Dengue is diagnosed by platelet count and/or symptoms due to lack of suitable infrastructure (IW, unpublished data).

#### Pucak

Pucak Village is situated in Tompu Bulu, Maros Regency, South Sulawesi Province (Fig. 1a, b, d). Approximately 31.9 km from the center of Makassar city, Pucak represents a rural area adjacent to Balai Taman Nasional Bantimurung Bulusaraung national forest. Pucak is approximately 1 km^2^ with a population of approximately 500 inhabitants and the main local economic activity is corn and rice farming. Here, the majority of housing consists of a one-room wooden construction on stilts, with permanent openings to the outside (open windows and doors and open-structured floor panels). Human excursions into the surrounding forested area represent points of contact between wildlife and humans. Pucak is considered to be malaria receptive. There are no data for dengue cases in Pucak.

### Study design

In both Lakkang and Pucak, collections were performed between April and May 2017, during the rainy season, for 2 weeks, on 4 consecutive nights each week (Table 1). Previous data collections (IW, unpublished data) in Lakkang and Pucak indicated primary mosquito activity between 18:00–00:00 h; therefore, collections were performed during this time. In Lakkang, there were three collection sites; Site 1 and 2 were outdoors, and Site 3 was indoors (Fig. 1c). Outdoor HLC and HDT trap locations were swapped on consecutive nights between Sites 1 and 2 in a cross-over design to prevent location bias (Table 1). In Pucak there were another three collection sites: Site 4 and Site 5 were outdoors, and Site 6 was indoors (Fig. 1d). Outdoor HLC and HDT trap locations here were switched on consecutive nights between Sites 4 and 5 (Table 1). Indoor HLCs were only performed at Sites 3 and 6 and their locations were not moved. Additionally, outdoor HLC and indoor HLC collectors switched locations every night after 3 h to remove collector bias. For all 6 collection nights in both villages environmental data, including nightly temperature, relative humidity, heat stress index, dew point, station pressure and density altitude were measured every minute using a Kestrel DROP D2 Wireless Temperature & Humidity Data Logger (www.kestrelinstruments.com).

**Table 1:** Collection study cross-over design for Lakkang and Pucak

Week	Site	Day 1	Day 2	Day 3	Day 4
Lakkang					
1	Site 1	HDT	Outdoor HLC	HDT	Outdoor HLC
	Site 2	Outdoor HLC	HDT	Outdoor HLC	HDT
	Site 3	Indoor HLC	Indoor HLC	Indoor HLC	Indoor HLC
2	Site 1	HDT	Outdoor HLC	HDT	Outdoor HLC
	Site 2	Outdoor HLC	HDT	Outdoor HLC	HDT
	Site 3	Indoor HLC	Indoor HLC	Indoor HLC	Indoor HLC
Pucak					
3	Site 4	HDT	Outdoor HLC	HDT	Outdoor HLC
	Site 5	Outdoor HLC	HDT	Outdoor HLC	HDT
	Site 6	Indoor HLC	Indoor HLC	Indoor HLC	Indoor HLC
4	Site 4	HDT	Outdoor HLC	HDT	Outdoor HLC
	Site 5	Outdoor HLC	HDT	Outdoor HLC	HDT
	Site 6	Indoor HLC	Indoor HLC	Indoor HLC	Indoor HLC

*Abbreviations*: HDT, host decoy trap; HLC, human landing catches

### Trap description

#### Human landing catches (HLCs)

HLCs were conducted as described by Gimnig et al. [35]. Collections were performed between 18:00 h and 00:00 h. There were four HLC collectors each night: two indoor and two outdoors. Collectors swapped positions halfway through the collection shift, at 21:00 h, to control for bias. Mosquitoes were placed in collection cups labelled according to the hour they were collected. Additionally, all collector positions were randomized at the start of each night. Outdoor HLCs were done to enable the comparison of outdoor HLC to the outdoor HDT, while indoor HLC collections enabled the comparison of indoor HLC to outdoor HLC.

#### Host decoy trap (HDT)

HDTs were produced by Biogents AG (Regensburg, Germany) based on previously described research [39] and prepared as described in Abong’o et al. [40]. The only deviation from this method was that the PVC pipe used to vent odors from the person in the tent was reduced from 10 m to 4.5 m in length. In summary, a collector slept in a one-person tent during the collection period (18:00 h to 00:00 h) and human odors from the tent were vented, *via* a pipe and fan, at the base of the HDT (Biogents). The HDT comprised a black cylinder, the surface of which was maintained at a temperature of 30–40 °C *via* hot water and wrapped in an adhesive plastic sheet (Barrettine Environmental Health, Bristol, UK). Mosquitoes are attracted to the host odors from the tent and induced to land by the visual and thermal properties of the HDT; they land and are trapped on the adhesive surface. At the end of every collection period, the adhesive sheet was removed, and trapped mosquitoes were extracted using glue board solvent (MobeMoat Solvent; Barrettine Environmental Health), with only very infrequent loss of body parts. Collections with HDT were performed at the outdoor collection sites only. HDT collections were done in 4 h shifts (18:00–00:00 h), with one collector inside the HDT tent per night. The single collector inside the HDT tent would rotate randomly on consecutive nights to remove collector bias.

### Mosquito collections

Collection village, site, date, collector identity, hour, genus, sex, and abdominal status were recorded for each mosquito collected by the HLC method. For each mosquito collected using the HDT, all the above data were recorded except for hour of collection. Mosquito morphological identifications were based on multiple taxonomic keys based on genera [44, 45]. For mosquitoes collected in Lakkang, at the end of every collection period, mosquitoes were stored individually in 1.5 ml Eppendorf tubes with silica desiccant for further processing. Mosquitoes collected in Pucak were dissected at the end of each collection period; the heads and thoraxes were stored individually in 1.5 ml Eppendorf tubes with 50 µl of RNAlater (Thermo Fisher Scientific, Waltham, USA) (for arbovirus identification) and the abdomens in 1.5 ml Eppendorf tubes with silica desiccant for further processing.

### Molecular processing

All *Anopheles* mosquitoes (*n* = 392) were sequenced at the mitochondrial DNA cytochrome *c* oxidase subunit 1 (*cox*1) locus for species identification. DNA was extracted from individual specimens using a modified version of the simple alkaline method by Rudbeck & Dissing [46]. Individual mosquitoes were ground in 1.5 ml tubes with 80 µl of 0.2 N NaOH and incubated at 75 °C for 10 min. After incubation, 29 µl 1M Tris, pH 8.0 and 891 µl of ddH_2_O was added and the tubes inverted 10 times. DNA was suspended in a final volume of 1000 µl containing 0.016 M NaOH and 0.029 M Tris-HCl, pH 8.0. The *cox*1 gene was amplified using LCO and HCO primers [47, 48]. Each reaction contained 1× Taq buffer (50 mM KCl, 10 mM Tris pH 9.0 and 0.1% Triton X), 1.5 mM MgCl_2_, 200 µM dNTPs, 5 pmol of each primer, 1 unit of *Taq* DNA polymerase, and 1 µl of genomic DNA as prepared above. Amplification was performed in 25 µl volumes in 96-well PCR plates (Dot Scientific, Burton, USA) in a Mastercycler Nexus thermocycler (Eppendorf, Hamburg, Germany). Thermocycling conditions for *cox*1 were as in Lobo et al. [47]. PCR products were size fractionated by electrophoresis in 2% agarose gels stained with SYBR™ Safe (Invitrogen, Carlsbad, CA, USA) and visualized under UV light. DNA cleanup and sequencing were conducted as in Lobo et al. [47] and St. Laurent et al. [48].

### Sequence analysis and species identification

Starting with a minimum match of 95%, the Seqman Pro Assembler (Lasergene v 10.1.1; www.dnastar.com) was used to align *cox*1 sequences. Using single nucleotide polymorphisms (SNPs), contigs were divided into sub-contigs. Consensus sequences were manually inspected for insertions, deletions, and repeat regions to ensure these sequence differences did not inflate divergence and decrease identity scores. *cox*1 sequences were assembled into final species identities using 95% identity, as mitochondrial lineages are more apt to diverge within a species [49, 50]. Species were identified by comparing (BLASTn and BOLD) consensus sequences of the *cox*1 contigs to the databases [51].

### Blood-meal analysis

Polymerase chain reaction (PCR) amplification of the mitochondrial *cytochrome b* gene was performed on all 163 blood-fed samples collected during the study. For amplification the following primer sets were utilized: L2513 (5′-GCC TGT TTA CCA AAA ACA TCA C-3′) and H2714 (5′-CTC CAT AGG GTC TTC TCG TCT T-3′) (~244 bp; see [52]); as well as L14841 (5′-CCA TCC AAC ATC TCA GCA TGA TGA AA-3′) and H15149 (5′-CCC TCA GAA TGA TAT TTG TCC TCA-3′) (~358 bp, see [53]). PCR conditions were the same as above. Thermocycling conditions were as follows: 95 °C for 5 min; 40 cycles of 94 °C for 30 s, 60 °C for 30 s, and 72 °C for 30 s; followed by an incubation at 72 °C for 5 min. PCR products were size fractionated by electrophoresis in 2% agarose gels stained with SYBR™ safe (Invitrogen) and visualized under UV light. DNA cleanup and sequencing were conducted as in Lobo et al. [47] and St Laurent et al. [48].

### Arboviruses identification

Heads and thoraxes of mosquitoes (*n* = 665 out of 672) from Pucak were tested for arboviruses. Individual specimens were pooled in groups of up to 30 based on genus to maximize efficiency: *Aedes*, *Anopheles*, *Armigeres* and *Culex*. A standard TRIzol RNA extraction as described by the manufacturer (Invitrogen) was used to isolate viral RNA. Extracted RNA was stored at − 20 °C until further testing. After RNA extraction, a reverse-transcriptase PCR was performed following the protocol for SuperScript III one-step RT-PCR System with Platinum *Taq* DNA Polymerase (Invitrogen) for all specimens using previously described primers: Flavivirus F (5′-TAC AAC ATG ATG GGA AAG AGA GAG AA-3′) and Flavivirus R (5′-GTG TCC CAG CCG GCG GTG TCA TCA GC-3′); Alphavirus F (5′-(CT)AG AGC (AGT)TT TTC GCA (CT)(GC)T (AG)GC (ACT) (AT)-3′) and Alphavirus R (5′-ACA T(AG)A AN(GT) GNG TNG T(AG)T C(AG)A ANC C(AGT)A (CT)CC-3′); and Bunyavirus F (5’-CTG CTA ACA CCA GCA GTA CTT TTG AC-3′) and Bunyavirus R (5′-TGG AGG GTA AGA CCA TCG TCA GGA ACT G-3′) [54–57].

### Statistical analysis

Statistical analyses were completed in R version 3.5.2 [58]. Catch abundance was analyzed using generalized linear models (GLMs; R package *MASS* [59]) with negative binomial distributions, which provided better fits to the 0-inflated data than models using Poisson distributions, followed by *post-hoc* Tukey comparisons between collection methods (R package *multcomp* [60]). When set as a predictor, collection site did not significantly affect catch (*χ*^2^ = 4.4209, *P* = 0.219) and so was not included as a factor in subsequent GLMs. HLC collectors working in pairs created a larger plume of CO_2_ and other olfactory cues, likely impacting mosquito numbers considerably [61]. Therefore, to normalize this result relative to the HDT, all nightly HLC collection results were divided by two, as collectors worked in pairs for HLC and singly for HDT. Sample size for indoor HLC *Anopheles* specimens was too low to examine biting activity in either Lakkang or Pucak individually.

## Results

A total of 2292 mosquitoes were collected in the study. Lakkang collections produced 1620 specimens, and Pucak 672. Across both study sites, the HDT collected 1361 mosquitoes, while the outdoor HLC collected 864 and the indoor HLC collected 67. Overall mean nightly catches were 85.1 ± 19.68 for the HDT, 27.3 ± 6.40 for the outdoor HLCs and 2.9 ± 0.88 for the indoor HLCs.

### Molecular species determination

The *cox*1 sequences of 382 *Anopheles* mosquito specimens were aligned into nine sequence groups with a stringency of greater than 95% identity within each group (Table 2). *Cox*1 database searches enabled identification of the following six sequence groups to species: *An. barbirostris*; *An. epiroticus*; *An. peditaeniatus*; *An. vagus*; *Cx. bitaeniorhynchus*; and *Ae. vexans* (Table 2). For *Anopheles* consensus sequence groups two, four, and five, BLASTn and BOLD sequence homology differed, and these groups were tentatively labelled *An. epiroticus/An. sundaicus* (*s.l.*), *An. nitidus/An. letifer*, and *An. nigerrimus/An. letifer*, respectively (Table 2).

**Table 2:** Overview of molecular identifications. Table represents the 382 morphologically identified *Anopheles* specimens that were sequenced using the *cox*1 locus. Final species identifications are based on both *cox*1 BLASTn and BOLD database comparisons

Consensus sequence groups	*n*	Collection method HDT/HLC	BLASTn sequence homology (%ID)	BOLD sequence homology (%ID)	Tentative species ID	Final species ID
Group 1	316	18/298	*An. barbirostris* (99)	*An. barbirostris* (98–96)	*An. barbirostris*	*An. barbirostris*
Group 2	32	4/28	*An. epiroticus* (97)	*An. sundaicus *(*s.l.*) (98–96)	*An. epiroticus/An. sundaicus* (*s.l.*)	Sundaicus Complex
Group 3	13	2/11	*An. epiroticus* (97)	*An. epiroticus* (97–96)	*An. epiroticus*	*An. epiroticus*
Group 4	7	0/7	*An. nitidus* (96)	*An. letifer* (98–95)	*An. nitidus/An. letifer*	Unknown
Group 5	5	0/5	*An. nigerrimus* (96)	*An. letifer* (98–95)	*An. nigerrimus/An. letifer*	Unknown
Group 6	3	0/3	*An. peditaeniatus* (99)	*An. peditaeniatus* (100–99)	*An. peditaeniatus*	*An. peditaeniatus*
Group 7	3	0/3	*An. vagus* (95)	*An. vagus* (99–90)	*An. vagus*	*An. vagus*
Group 8	2	1/1	*Cx. bitaeniorhynchus* (100)	*Cx. bitaeniorhynchus* (100–94)	*Cx. bitaeniorhynchus*	*Cx. bitaeniorhynchus*
Group 9	1	0/1	*Ae. vexans* (99)	*Aedimorphus vexans* (99–97)	*Ae. vexans*	*Ae. vexans*

*Abbreviations*: %ID, the percentage identity based on BLASTn and BOLD database comparisons, n, number of samples analyzed

In Lakkang, 7 species of *Anopheles* mosquitoes (*n* = 373) were collected. *Anopheles barbirostris* made up the majority of the collection (83.2%; 311/373), with smaller proportions of *An. epiroticus/An. sundaicus* (*s.l.*) (8.6%; 32/373), *An. epiroticus* (3.5%; 13/373), *An. nitidus/An. letifer* (1.9%; 7/373), *An. nigerrimus/An. letifer* (1.3%; 5/373), and < 1 of both *An. peditaeniatus* (0.8%; 3/373) and *An. vagus* (0.5%; 2/373). Three specimens were morphologically misidentified as *Anopheles* and consequently molecularly verified as *Cx. bitaeniorhynchus* and *Ae. vexans*. Outdoor HLC collected all seven species of *Anopheles* mosquitoes in Lakkang as well as the single *Ae. vexans* specimen. Indoor HLC and HDT collected three of these species of anophelines: *An. barbirostris*, *An. epiroticus/An. sundaicus* (*s.l.*), and *An. epiroticus*.

In Pucak, two species of *Anopheles* mosquitoes (*n* = 6) were collected, *An. barbirostris* (*n* = 5) and *An. vagus* (*n* = 1). Both of these species were collected using outdoor HLC. Two morphologically identified *Anopheles* specimens were molecularly identified as *Cx. bitaeniorhynchus*. These *Culex* specimens were collected with HDT and indoor HLC.

### Nightly catch comparison by collection method

#### Lakkang

**Fig. 2:**
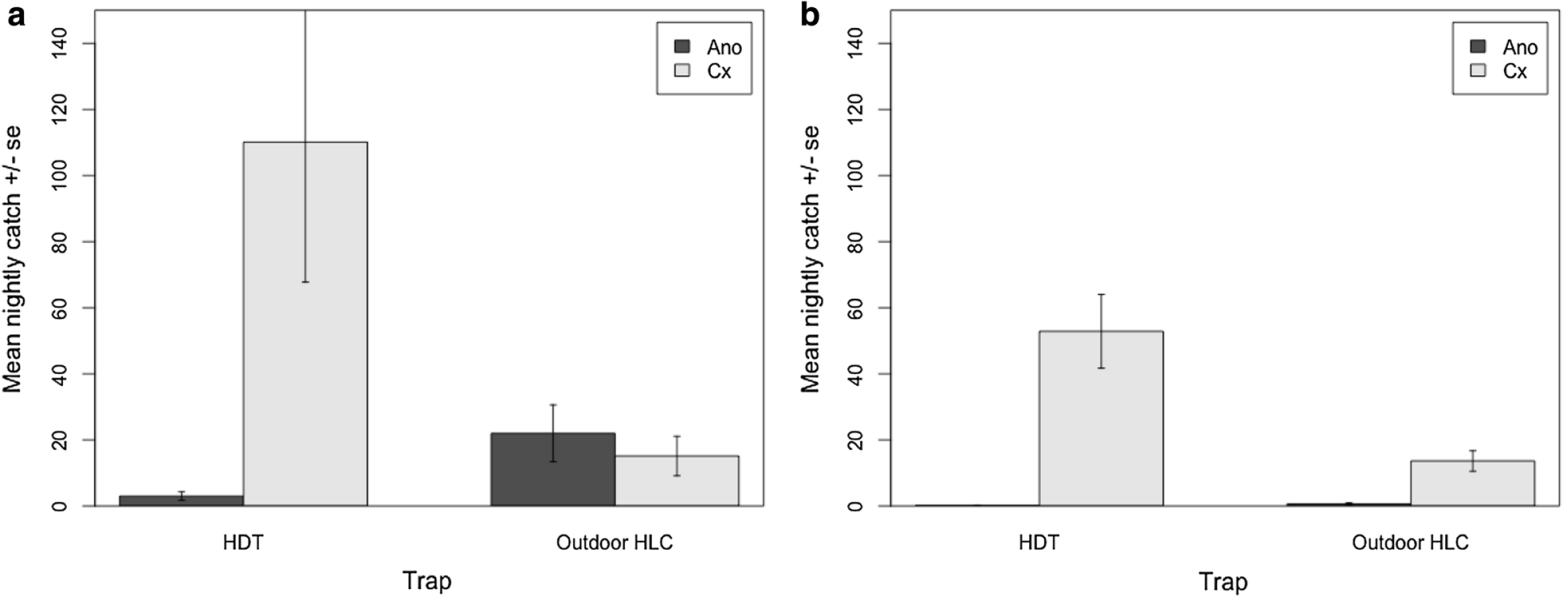
Nightly catch (mean ± standard error) of anopheline (Ano) and culicine (Cx) mosquitoes from human decoy trap (HDT) and outdoor human landing catch (HLC) traps in Lakkang (**a**) and Pucak (**b**), Sulawesi, Indonesia

Mosquito abundance in Lakkang differed by trap type; more *Anopheles* were collected by the outdoor HLC, while the HDT collected a greater abundance of *Culex* mosquitoes. For *Anopheles*, the outdoor HLCs collected significantly more mosquitoes per night (22.0 ± 8.6; *F*_(1, 14)_ = 23.275, *P *< 0.0001) than the HDT (3.0 ± 1.3), representing a seven-fold difference (Fig. 2a). Meanwhile, the HDT collected a significantly greater nightly average of 110.1 ± 42.4 *Culex* mosquitoes, over seven times more compared to the outdoor HLC nightly average of 15.1 ± 6.0 (*F*_(1, 14)_ = 8.1756, *P* = 0.004) (Fig. 2a). Indoor HLCs were not statistically compared to other trapping methods due to low sample size of total indoor catches in Lakkang (*n* = 43).

#### Pucak

Mosquito abundance in Pucak also differed by trap type; the HDT again collected a greater abundance of *Culex* collections compared to the HLC. For *Anopheles*, the HDT collected a nightly mean of 0.1 ± 0.1 mosquitoes and there was no significant difference in this compared to outdoor HLC, which caught a mean of 0.6 ± 0.3 per night (*F*_(1, 14)_ = 2.9109, *P* = 0.088) (Fig. 2b).The HDT collected a nightly average of 52.9 ± 11.2, significantly more *Culex* mosquitoes compared to the outdoor HLC nightly average of 13.6 ± 3.1 (*F*_(1, 14)_ = 17.137, *P *< 0.0001) (Fig. 2b). Indoor HLCs were not statistically compared to other trapping methods due to low sample size of total indoor catches in Pucak (*n* = 24).

### Genera composition by collection method

**Fig. 3:**
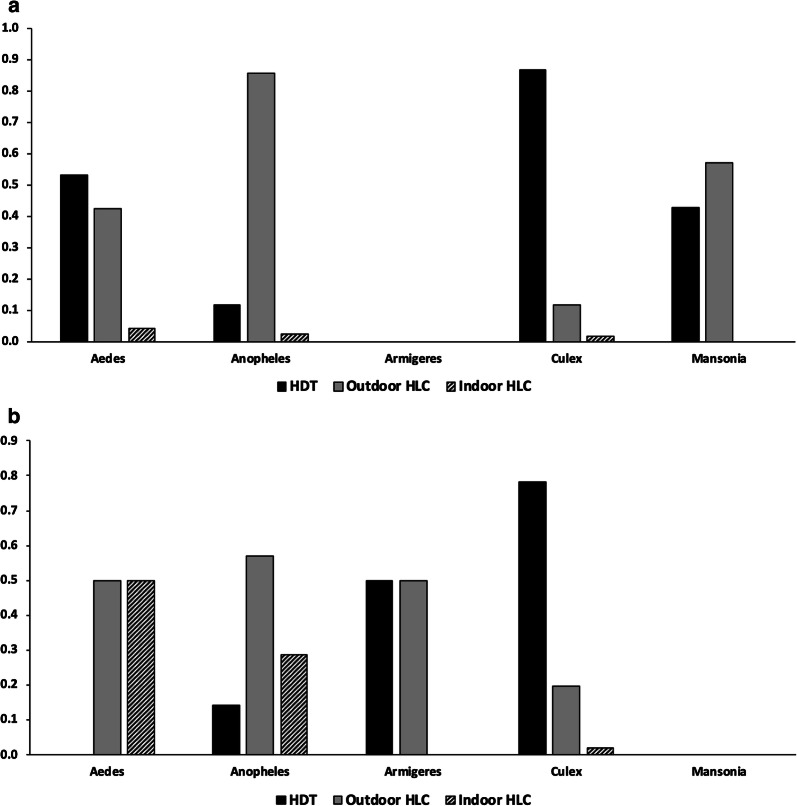
Comparison of three trapping methods, human decoy trap, outdoor HLC, and indoor HLC by proportion of total mosquitoes collected within a given genus in Lakkang (**a**) and Pucak (**b**) Sulawesi, Indonesia

**Fig. 4:**
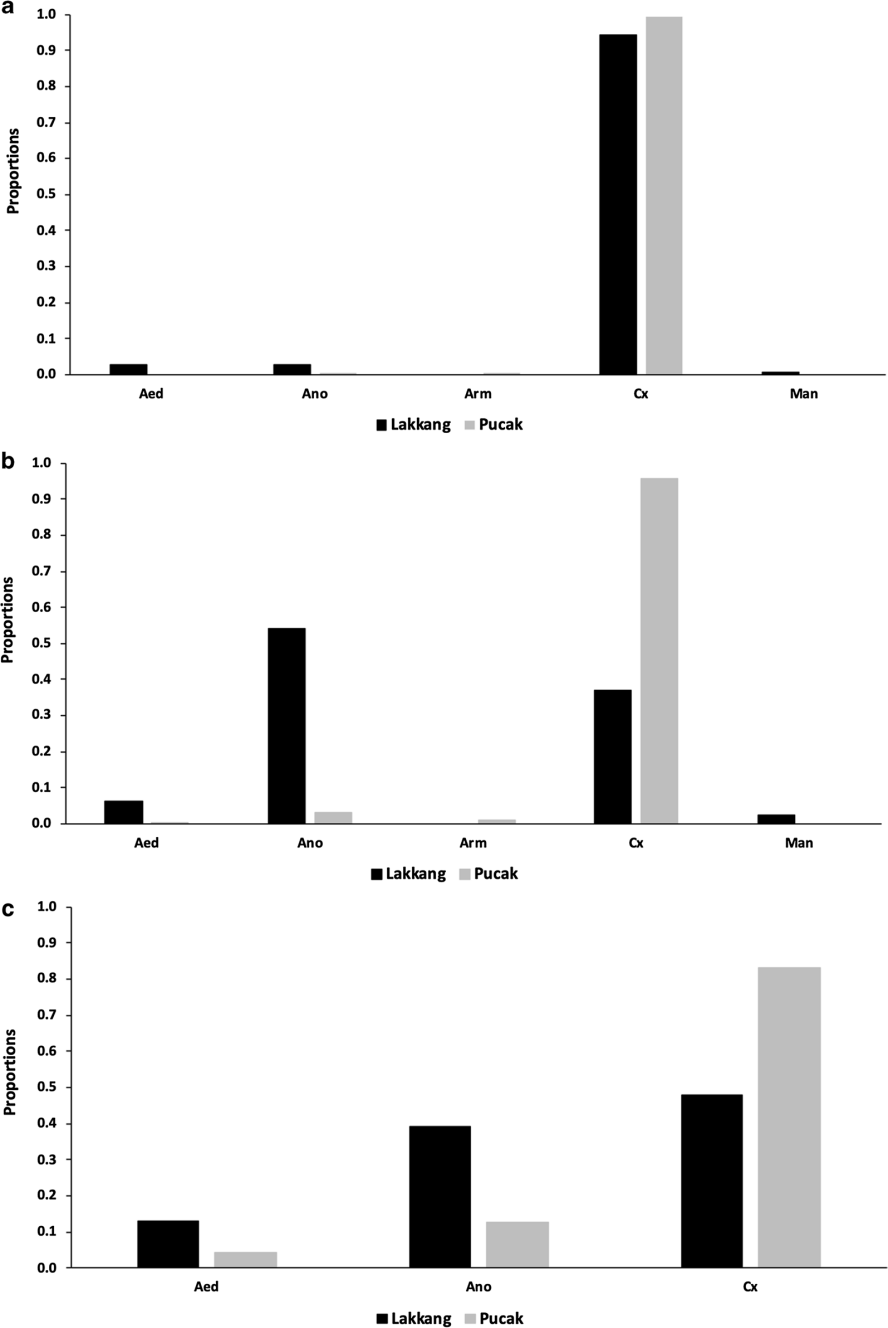
Relative genera compositions (proportions) of *Aedes* (Aed), *Anopheles* (Ano), *Armigeris* (Arm), *Culex* (Cx), and *Mansonia* (Man), from three trapping methods, (**a**) human decoy trap (*n* = 1361), (**b**) outdoor human landing catch (*n* = 864) and (**c**) indoor human landing catch (*n* = 67) in Lakkang and Pucak, Sulawesi, Indonesia

First, trap collections were compared by the proportion of a given genus out of total collections of that genus at each site. In Lakkang, the majority of *Anopheles* specimens were collected with outdoor HLCs, 85.7% (Fig. 3a). The HDT and indoor HLCs collected 11.8% and 2.5% of *Anopheles*, respectively (Fig. 3a). The majority of *Culex* specimens were collected with the HDT, 86.7%. Outdoor HLCs and indoor HLCs collected 11.7% and 1.6% of *Culex* mosquitoes, respectively (Fig. 3a). *Aedes* species collected with the HDT, outdoor HLC and indoor HLC consisted of 53.2%, 42.6% and 4.3%, respectively (Fig. 4a). Outdoor HLCs collected over half of *Mansonia* mosquitoes 57.1% with HDTs collecting the remaining 42.9% of *Mansonia* specimens (Fig. 3a). There were no *Armigeres* mosquitoes collected in Lakkang (Fig. 3a).

In Pucak, outdoor HLCs collected of half of *Anopheles* species in Pucak, 57.1%, followed by indoor HLC, 28.6%, then the HDT 14.3% (Fig. 3b). The HDT again collected the majority of the total *Culex* mosquito collection (78.3%), while outdoor HLCs and indoor HLCs collected 19.8% and 1.9%, respectively (Fig. 3b). Outdoor and indoor HLCs each collected 50% of *Aedes* species in Pucak while the HDT and outdoor HLC each collected 50% of *Armigeres* mosquitoes (Fig. 3b). There were no *Mansonia* mosquitoes collected in Pucak (Fig. 3b).

Secondly, traps were evaluated by looking at a single trapping method’s rate of collection of a genus compared to other genera. For instance, the overwhelming majority of mosquitoes collected by the HDT were *Culex* in both Lakkang (94.1%) and Pucak (99.5%), with 2.6% *Anopheles*, 2.7% *Aedes*, and 0.6% *Mansonia* in Lakkang and 0.2% *Anopheles* and 0.2% *Armigeris* in Pucak (Fig. 4a). This differed markedly to the genera composition from outdoor HLC, for which *Anopheles* dominated in Lakkang (54% of the catch) but accounted for only 3.1% of the catch in Pucak (Fig. 4b). There was, however, a similar proportion of *Culex* collected by the outdoor HLC in Pucak (95.5%) compared to Pucak HDT (99.5%). Indoor HLCs did not collect any *Armigeris* or *Mansonia* mosquitoes. There were similar proportions of *Anopheles* (20.9%) and *Culex* (72.1%) in Lakkang, but indoor HLC had a higher proportion of *Culex* (83.3%) compared to *Anopheles* and *Aedes* in Pucak (Fig. 4c).

### Blood-meal identification and abdominal status

**Fig. 5:**
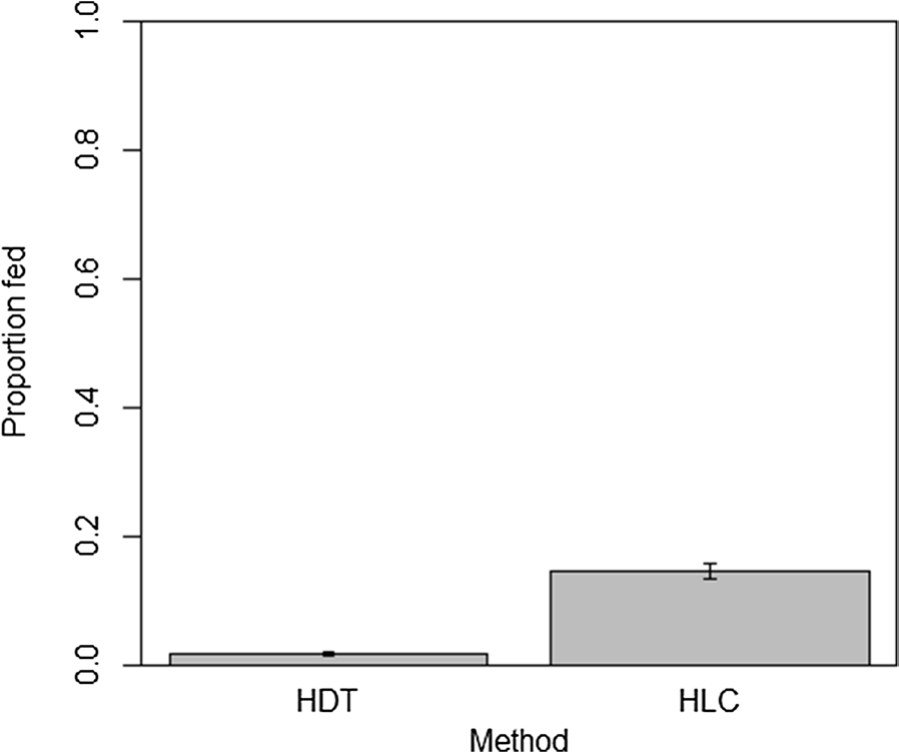
Proportion of blood-fed mosquitoes collected with the host decoy trap (HDT) compared to outdoor human landing catch (HLC) for the duration of the study

Outdoor HLCs collected a significantly higher proportion of blood-fed mosquitoes (66/433) compared to the HDT (23/1360), 0.15 ± 0.02 and 0.02 ± 0.0 (*z* = 9.329, *P* < 0.0001) (Fig. 5). Blood-fed culicines collected with the HDT (20/1304) and outdoor HLC (36/226) differed significantly (*z* = 8.63, *P* < 0.001). Whereas, *Anopheles* blood-fed proportions between the HDT (1/25) and outdoor HLC (18/178) were not significantly different (*z* = 0.946, *P* = 0.975).

Engorged females (*n* = 163) were tested for blood meal by PCR to identify the host source (Table 3). For the HDT (*n* = 19), 52.6%, 36.8% and 10.5% of identified blood meals were on cows, humans and dogs respectively. For mosquitoes collected with the HDT, a single *Aedes* and a single *Anopheles* specimen fed on cows, while the only blood-fed *Mansonia* mosquito fed on humans (Table 3). The remaining blood-fed *Culex* mosquitoes collected with the HDT fed on cows, humans and dogs (*n* = 8, 6 and 2, respectively). Identified blood meals for outdoor HLCs (*n* = 111) were 91.9% human, 6.3% cow, and 0.9% each dog and cat. *Aedes* mosquitoes collected with outdoor HLC had fed on humans and cows (*n* = 7 and *n* = 1, respectively), as had *Anopheles* mosquitoes (*n* = 32 and *n* = 2, respectively). *Culex* mosquitoes collected with outdoor HLC fed on humans, cows, dogs, and cats (*n* = 58, 4 1 and 1) respectively (Table 3). The *Mansonia* mosquitoes collected with outdoor HLC only fed on humans (*n* = 5). Of the *Anopheles* species that were molecularly identified, 39 had identified blood meals. *Anopheles barbirostris* (29/39) blood-fed on humans and cows (*n* = 26 and *n* = 3), respectively. *Anopheles epiroticus/An. sundaicus* (*s.l.*) (5/39), *An. epiroticus* (3/39), *An. nitidus/An. letifer* (1/39) and *An. peditaeniatus* (1/39) all blood-fed on humans.

**Table 3:** Number of blood-fed specimens collected and successful host DNA identification groups based on *cytochrome b* gene analysis

Morphological genera/collection method	No. of specimens collected	No. of blood meals identified	Human	Cow	Dog	Cat
HDT						
*Aedes*	1	1	0	1	0	0
*Anopheles*	1	1	0	1	0	0
*Culex*	20	16	6	8	2	0
*Mansonia*	1	1	1	0	0	0
Total	23	19	7	10	2	0
Outdoor HLC						
*Aedes*	9	8	7	1	0	0
*Anopheles*	35	34	32	2	0	0
*Culex*	71	64	58	4	1	1
*Mansonia*	11	5	5	0	0	0
Total	126	111	102	7	1	1

*Abbreviations*: HDT, host decoy trap; No., number; HLC, human landing catch

### Arbovirus identification

Mosquitoes from Pucak tested for arboviruses indicated one *Culex* pool positive for flavivirus, one *Anopheles* pool positive for alphavirus, and one *Armigeres* pool positive for flavivirus.

### Biting activity

**Fig. 6:**
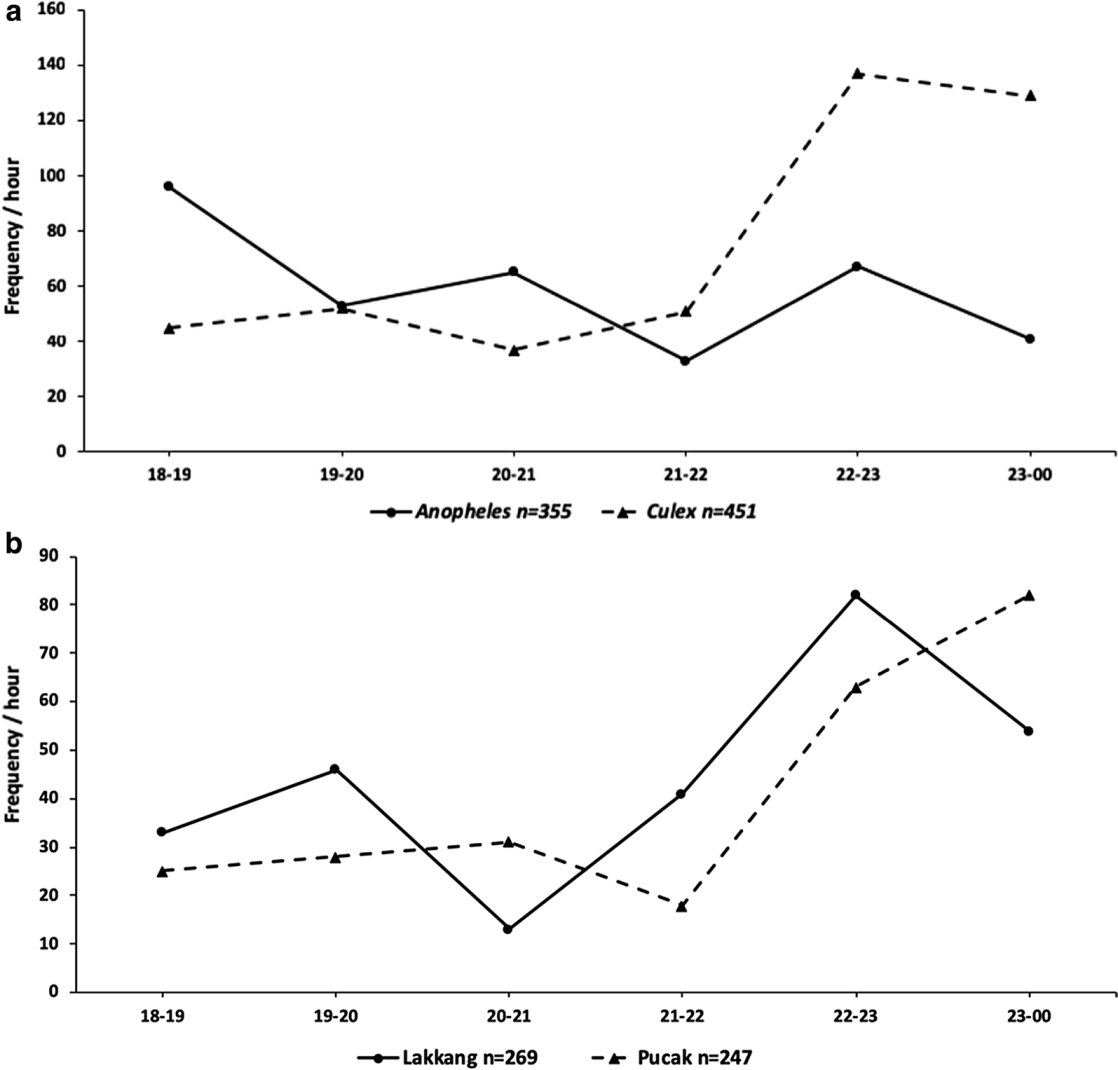
Biting activity by hour for outdoor HLC for *Culex* and *Anopheles* specimens (**a**) and *Culex* specimens in Lakkang and Pucak (**b**). Frequency was calculated as the number of mosquitoes collected utilizing outdoor HLC for each time point throughout the first half of the night

Overall biting activity of *Anopheles* peaked between 18:00–19:00 h with smaller subsequent peaks between 20:00–21:00 h and 22:00–23:00 h (Fig. 6a). Overall biting activity of *Culex* mosquitoes peaked from 22:00–23:00 h (Fig. 6a). *Culex* mosquito biting activity in Lakkang experienced a small peak in activity between 19:00–20:00 h followed by a decrease in activity between 20:00–21:00 h (Fig. 6b). Biting activity of *Culex* mosquitoes rose between 21:00–23:00 h before decreasing (Fig. 6b). In Pucak biting activity of *Culex* mosquitoes experienced a small peak between 20:00–21:00 h followed by a decrease in activity between 21:00–22:00 h (Fig. 6b). Biting activity of *Culex* mosquitoes increased from 22:00–00:00 h, during the second half of the night (Fig. 6b).

### Environmental data

Average nightly temperature and relative humidity (RH) in Lakkang were 27.5 °C and 80.8% RH, and in Pucak were 26.4 °C and 85.6% RH (Table 4).

**Table 4:** Kestrel DROP D2 Wireless temperature and humidity data

Village	Temperature (°C)	Relative humidity (%)	Heat stress index (°C)	Dew point (°C)	Station pressure (mb)	Density altitude (m)
Lakkang	27.5	80.8	31.5	23.8	1010.2	584.7
Pucak	26.4	85.6	30.2	23.8	998.6	669.9

*Abbreviations*: mb, millibars

## Discussion

This study found that the HLC caught significantly more *Anopheles* mosquitoes than HDT, in contrast to results from the HDT pilot study [39]. Conducted in Burkina Faso, Africa, the pilot study consisted of catches from predominantly one anopheline species, *An. coluzzii*, and found that HDT collections surpassed HLC for all mosquito genera [39]. In this study, outdoor HLCs surpassed HDTs for *Anopheles* in Lakkang, suggesting that behaviors displayed by African *Anopheles* vectors may be drastically different than those shown by Indonesian *Anopheles* vectors. This stresses the need for continued laboratory and field studies to test the responses of different anophelines to specific cues. Only by doing so can the nuances of behaviors be understood and targeted in a trap like the HDT, providing the evidence-base for locally appropriate innovations in surveillance and control.

The difference in collection performance may be due to the specific behavioral traits of local anopheline species. Based on our results, host-seeking behaviors in Indonesian anophelines are presumably sufficiently different to those of the Gambiae Complex species to lead to differential responses to the host-associated cues used in the HDT. Inclusion and configuration of these cues in trap design has been based on both laboratory behavioral assays and subsequent field-based proof of concept studies [39] that have concentrated on *An. gambiae* (*s.l*.). As the HDT was effective at sampling culicines (both in comparison to HLC within this study and relative to previous results), it therefore seems likely that the difference in catch stems from fundamental differences between Indonesian and African anopheline behavior. This challenges the notion that ‘universal’ tools for sampling or controlling diverse secondary malaria vectors will be effective, implying instead that tailored solutions, adapted to the specific behavior and ecology of local vectors, will be needed. Such tools will require empirical study of these traits in less well understood vector species to provide the basis for locally effective innovations.

St. Laurent et al. [62] identified similar anopheline communities in Cambodia to those found in the present study in Sulawesi, including *An. vagus*, *An. nitidus*, *An. peditaeniatus* and the Barbirostris group. In Thailand, *An. epiroticus* displayed exophagic and zoophilic preferences and the majority of these species were collected in buffalo traps compared to HLC [63]. A study examining host preferences of anophelines in Sri Lanka [64] did not detect any human blood in two species identified in the present study, i.e. *An. nigerrimus* and *An. vagus*. Additionally, the human blood index calculated for *An. barbirostris* was 0.27, indicating lower human blood meal preference compared to other vectors in the region [64]. Further research will be needed to investigate the strength of this relationship.

The HDT, as configured in this study, appears to be effective for collecting all other mosquito genera, *Culex*, *Mansonia* and *Armigeres*, collecting significantly more *Culex* mosquitoes and similar amounts of other genera. Existing sampling methods for these groups have been tested and found to be effective in the Southeast Asian region, including CDC light traps [65] and tent traps baited with various animal baits [66]. These tend to require less equipment than the current HDT prototype and this would require further product development to improve the practicality of the tool before being considered a viable field method.

Despite their importance in transmitting filariasis [67, 68], Japanese encephalitis [69–71], and other lesser known arboviruses [26, 69, 72, 73], little is known about the distribution and bionomics of culicine vectors in Sulawesi, Indonesia. Furthermore, there have been several arboviruses outbreaks in recent Indonesian history including chikungunya virus [12, 74], and the isolation of Zika virus from Jakarta and Bali [66, 75]. Future studies could utilize the HDT for identifying potential vector-borne pathogen exposure risks. This study serves as proof of principle for utilizing the HDT for culicine sampling and surveillance in this region.

Inarguably, outdoor HLCs are optimized for human host-seeking anophelines, as supported by the results of this study and previous literature [76]. Although the previously mentioned HDT studies had success using other configurations, namely cow-baited decoy traps, at collecting *Anopheles* vectors that feed on humans, the method is still limited in how closely it can mirror outdoor HLCs. Although the risks of utilizing willing, trained volunteers providing informed consent to perform HLCs are widely accepted due to the otherwise impossible to approximate epidemiological risk factors, research demonstrating configurations of the HDT that more closely mirror HLC collections could potentially eliminate this risk. Although this study failed to demonstrate the ability for the HDT to mimic HLCs, it does demonstrate some of the risk involved; outdoor HLCs collected more blood-fed mosquitoes than HDTs in both Lakkang and Pucak, the majority of this difference is based almost entirely on human blood-fed mosquitoes, which comprised 91.9% of outdoor HLC blood-fed mosquitoes. Since the HDT protects collectors from mosquito bites and only uses their odors to attract mosquitoes, a possible reason for the majority of human blood meals (and therefore total blood meals) were mosquitoes that fed on HLC collectors. This discrepancy could also be due to the different mosquito compositions collected. Further studies, involving matching DNA from blood-fed mosquitoes to collectors could establish the strength of the relationship between HLCs and collector exposure through comparison with HDT blood-fed rates. Given HLCs accounted for a disproportionately high number of blood-fed mosquitoes, their use in entomological studies when risk of arboviral transmission is present should be avoided where possible.

In this study, two pools of mosquitoes tested positive for flavivirus, one *Culex* and one *Armigeres* while one *Anopheles* pool tested positive for alphavirus, highlighting the potential risk to collectors. While there are no reports of any arboviruses in circulation in human populations in Pucak, local diagnostic capacity may limit accurate detection and reporting of these. Viral transmission may be occurring since viruses are present in the mosquito vectors.

Based on the HLC results, this study was able to characterize *Anopheles* species in a region without previous collection. Of the nine molecularly identified consensus sequences, six could be identified to species: *An. barbirostris*; *An. epiroticus*; *An. peditaeniatus*; *An. vagus*; *Cx. bitaeniorhynchus*; and *Ae. vexans*. Of the four molecularly identified *Anopheles*, three are confirmed malaria vectors in Indonesia: *An. barbirostris*; *An. epiroticus*; and *An. vagus* [7, 77–80]. *Anopheles peditaeniatus* has not been confirmed as a malaria vector in Indonesia; however, it has been shown positive for *Plasmodium falciparum* by ELISA in Sri Lanka [81] and Thailand [82]. The presence of these species in human-baited traps supports the description of both Makassar and Maros Regencies as malaria receptive areas by regional public health teams (IW, unpublished data). Three specimens were morphologically identified as *Anopheles* but molecularly confirmed to be *Cx. bitaeniorhynchus* and *Ae. vexans*. *Culex bitaeniorhynchus* is a vector of *Wuchereria bancrofti*, Murray Valley encephalitis virus, Japanese encephalitis virus, and Batai virus [83–86], while *Ae. vexans* is capable of transmitting Eastern equine encephalitis virus, Western equine encephalitis virus, St. Louis encephalitis virus, West Nile virus, and Japanese encephalitis [87–89]. This finding highlights the importance of cross-referencing morphological identifications with molecular identifications, especially in areas of high vector diversity, like Indonesia. Misidentifications resulting from overreliance on a single identification method can have negative downstream effects when determining species’ bionomic traits, associations of vector status, entomological inoculation rates, and impacts of control. Inaccurate morphological identification at the genus level stresses the need for employing molecular identification in otherwise morphology-based studies when determining disease vectors and their respective bionomics.

While molecular tools can disambiguate vector identities, they must be based on comprehensive libraries of reference genetic material. In our study, three consensus sequences had conflicting BLASTn and BOLD database results: group 2, 4, and 5. Group 2 belongs to the Sundaicus Complex, a main malaria vector in Indonesia [7]. Group 4 BLASTn database homology identified these specimens as *An. nitidus*; however, the BOLD database search identified these *Anopheles* specimens as *An. letifer*. The discrepancy in the BLASTn and BOLD database sequence results coupled with the presence of *cox*1 sequences for *An. letifer* in BLASTn and *An. nitidus* in BOLD, and lack of associated sequences in databases could indicate an addition to the Hyrcanus or Umbrosus Group. However, it is far more likely that distributions and phylogenetic relationships between species in these two groups may vary due to the wide geographical distribution of sequences available in databases, complicating interpretation. For example, the *An. letifer* sequences in GenBank are from Singapore [90], while these *An. nitidus* sequences in BOLD are from Thailand. Similar results were seen for group 5. Therefore, additional research that implements nuclear and mtDNA sequencing within Indonesia is necessary to accurately identify species that are malaria and arboviral vectors and to create baseline information for nucleotide databases for Sulawesi specifically.

Local *Anopheles* vector populations were composed of up to seven species, consisting of predominantly *An. barbirostris*. Seven different *Anopheles* species were identified in Lakkang, whereas only two *Anopheles* species were found in Pucak. The diversity in mosquito vectors in two 1 km^2^ villages further illustrates the biodiversity of the Indonesian archipelago and its ability to host a variety of vectors. Appropriate innovations in surveillance and control, especially in regions of high biodiversity with heterogenous vector ecology and behavior, are undoubtedly a challenge [4, 91].

Genera composition varied between Lakkang and Pucak. *Aedes*, *Anopheles* and *Mansonia* were found in higher proportions in Lakkang, while *Culex* and *Armigeres* were found in higher proportions in Pucak. These differences are attributed to the presence of available habitats preferred by species of these genera. Lakkang is surrounded on all sides by the Tallo River and the main economic activity is rice farming. Due to its riverine location and predominance of rice paddy land cover, sunlit water bodies are readily available, making the surrounding area an excellent habitat for many *Anopheles* species, including the three main species found in this study, namely *An. barbirostris*, *An. epiroticus* and *An. sundaicus* (*s.l.*) [92–94]. Whereas, south of the collection sites in Pucak, there is water run-off from the neighboring river. The resulting pools of often stagnant and turbid water are an ideal habitat for *Culex* larvae [95]. More generally, in North Sulawesi, studies have reported a *Culex* preference for rain-fed rice fields [96]. The rice fields in both Lakkang and Pucak are rain-fed and this could explain the high proportions of *Culex* specimens relative to *Anopheles* specimens at these study sites. However, this could also be a result of zoophilic *Anopheles* being present at the sites that were not targeted by the trapping regimen in this study. Lastly, previous studies have shown an increase in the host-seeking behaviors of *An. gambiae* (*s.s.*) and an increase in *Mansonia* mosquito populations during periods of rising relative humidity [97, 98]. The higher relative humidity in Pucak with the available bodies of water for *Culex* larvae could explain the elevated numbers of *Culex* mosquitoes collected in Pucak, however future studies would need to further investigate this relationship.

Indoor and outdoor mosquito biting profiles were compared with HLC. Indoor HLCs collected low proportions of all mosquito genera in both villages compared to outdoor HLCs, despite the predominantly open housing structures found in both study villages. Given there are limited physical barriers to impede mosquito ingress into dwellings, these low numbers indicate mosquito biting is preferentially occurring outdoors, suggesting implementation of outdoor interventions may be most effective in both Lakkang and Pucak, Indonesia, if disease transmission is confirmed in this susceptible region.

Biting activity is an important factor for determining the behaviors and possible transmission times for vector-borne diseases. *Anopheles* mosquitoes peaked in activity between 18:00–19:00 h. This corroborates previous reports of *An. barbirostris*, the predominate molecularly identified vector in this study, that indicate *An. barbirostris* feeds outdoors in the early evening [99]. However, the biting activity for *An. barbirostris* are dependent on geographical location in Indonesia [7]. There have been no attempts to evaluate the biting activity of *Culex* mosquitoes in Lakkang or Pucak. In both villages, *Culex* activity increased during the second half of the night peaking at 22:00–23:00 h in Lakkang and continuing to rise up to the end of collections at 00:00 h in Pucak. Although collections in this study represent a fourth of the daily timeline, it aligns with other field studies from India and Thailand that have reported peaks in *Cx. quinquefasciatus* flight activity around 22:00 h [100, 101]. In Japan, the average density of *Cx. pipiens* was found to increase until 22:00 h [102]. A limitation of this study was that molecular identification was not performed on culicine mosquitoes. The lack of *Culex* biting activity documentation in Indonesia stresses the importance of continued recording of mosquito biting times so accurate local transmission risks can be determined.

Identifying and evaluating species compositions and behaviors in Indonesia will require continued interest and investment from researchers. The region’s high biodiversity and archipelagic geography make efforts to comprehensively survey the islands either prohibitively expensive or piecemeal and individualized to localities. Furthermore, behavior of local vector fauna differs markedly to the relatively well-studied African vectors. Combining longitudinal data sets for trap types and comparison studies will yield better understanding for ethical trapping and effective intervention practices as the world continues to pursue the elimination of malarial and arboviral transmission.

## Conclusions

Although the HDT collected the highest density of mosquitoes in this study, this was entirely due to culicine specimens, which were not evaluated at the species level. Outdoor HLCs collected the highest abundance of anopheline specimens. Therefore, although the stimuli presented in the HDT can be employed to sample a range of different Asian mosquito genera and species, it remains to be optimized for specific host-seeking behaviors in Asia. The high proportion of human blood meals from mosquitoes collected by outdoor HLCs reinforces what is known about the exposure risk to collectors for the methodology and the importance of continuing to optimize a host-mimic trap such as the HDT. Further study involving matching DNA from blood-fed mosquitoes to collectors could establish the strength of the relationship between HLCs and collector exposure, especially through comparison with mosquito blood-meals of a host-mimic trap, such as an optimized HDT. The HDT could also be evaluated as a potential outdoor intervention strategy if the high capture rates can be demonstrated to impact overall mosquito populations within a locality.

## Data Availability

Data supporting the conclusions of this article are included within the article. Raw data used and/or analyzed during the present study are available from the corresponding author upon reasonable request.

## Abbreviations

HDT: host decoy trap
HLC: human landing catch
ITN: insecticide-treated net
GLM: generalized linear model
PCR: polymerase chain reaction
